# Social representation of young people in higher education about sexually transmitted infections

**DOI:** 10.1590/0034-7167-2022-0406

**Published:** 2023-12-04

**Authors:** Thelma Spindola, Laércio Deleon de Melo, Juliana de Lima Brandão, Denize Cristina de Oliveira, Sérgio Corrêa Marques, Cristina Arreguy-Sena, Paulo Ferreira Pinto

**Affiliations:** I Universidade do Estado do Rio de Janeiro. Rio de Janeiro, Rio de Janeiro, Brazil; II Universidade Federal de Juiz de Fora. Juiz de Fora, Minas Gerais, Brazil

**Keywords:** Young Adult, Education, Higher, Sexually Transmitted Diseases, Social Representation, Public Health., Adulto Joven, Educación Superior, Enfermedades de Transmisión Sexual, Representación Social, Salud Pública., Adulto Jovem, Educação Superior, Infecções Sexualmente Transmissíveis, Representação Social, Saúde Pública

## Abstract

**Objective:**

to analyze the social representations about sexually transmitted infections elaborated by undergraduate students.

**Methods:**

a descriptive, qualitative study, in the light of the structural approach of Social Representation Theory, carried out with 160 young undergraduate students, in the second half of 2019, in the city of Rio de Janeiro. Data were collected using a sociodemographic characterization questionnaire, knowledge and practices for preventing sexually transmitted infections, analyzed using descriptive statistics and a form of free evocations with the inducing term STD, analyzed using prototypical and similarity analysis.

**Results:**

the representation’s possible central nucleus is composed of lexicons aids, disease and HIV; the peripheral system by syphilis, sex, condoms, gonorrhea, prevention, infection, carelessness, HPV, herpes, ignorance, treatment, fear, unprotected-sex and danger.

**Final considerations:**

social thinking about sexually transmitted infections is characterized by their recognition as diseases, which require barrier prevention measures, associating with unsafe sexual practices that arouse fear.

## RESUMO

**Objetivo:**

analisar as representações sociais sobre as infecções sexualmente transmissíveis elaboradas por jovens universitários.

**Métodos:**

estudo descritivo, qualitativo, à luz da abordagem estrutural da Teoria das Representações Sociais, realizado com 160 jovens universitários, no segundo semestre de 2019, na cidade do Rio de Janeiro. Os dados foram coletados por questionário de caracterização sociodemográfica, conhecimentos e práticas de prevenção das infecções sexualmente transmissíveis, analisados pela estatística descritiva e por formulário de evocações livres com o termo indutor DST, analisadas pela análise prototípica e de similitude.

**Resultados:**

o possível núcleo central da representação é composto pelos léxicos aids, doença e HIV; o sistema periférico, por sífilis, sexo, camisinha, gonorreia, prevenção, infecção, descuido, HPV, herpes, desconhecimento, tratamento, medo, sexo-sem-proteção e perigo.

**Considerações finais:**

o pensamento social sobre as infecções sexualmente transmissíveis caracteriza-se pelo seu reconhecimento como doenças que requerem medidas de prevenção de barreira, e associam-se a práticas sexuais inseguras que despertam medo.

## RESUMEN

**Objetivo:**

analizar las representaciones sociales sobre las infecciones de transmisión sexual elaboradas por estudiantes universitarios.

**Métodos:**

estudio descriptivo, cualitativo, a la luz del enfoque estructural de la Teoría de las Representaciones Sociales, realizado con 160 jóvenes universitarios, en el segundo semestre de 2019, en la ciudad de Río de Janeiro. Los datos fueron recolectados mediante un cuestionario de caracterización sociodemográfica, conocimientos y prácticas para la prevención de infecciones de transmisión sexual, analizados mediante estadística descriptiva y un formulario de evocaciones libres con el término inductor de ETS, analizados mediante análisis prototípico y de similitud.

**Resultados:**

el posible núcleo central de la representación está compuesto por los léxicos sida, enfermedad y VIH; el sistema periférico, por sífilis, sexo, condones, gonorrea, prevención, infección, descuido, VPH, herpes, desconocimiento, tratamiento, miedo, sexo sin protección y peligro.

**Consideraciones finales:**

el pensamiento social sobre las infecciones de transmisión sexual se caracteriza por su reconocimiento como enfermedades que requierendo medidas de prevención de barrera, se asociando a prácticas sexuales inseguras que despiertan miedo.

## INTRODUCTION

Sexually transmitted infections (STIs), better known socially as sexually transmitted diseases (STDs), still represent a public health problem of global contingency^(1-4)^. A significant increase in new cases of STIs is observed in Brazil, with a higher occurrence among young people aged 15 to 29 years, mainly related to HIV and syphilis^(4)^.

Young people are considered a vulnerable social group to acquire STIs, as they have sexual behaviors that predispose to the acquisition of STIs, such as early initiation of sexual life, discontinuous or incorrect use of condoms, occurrence of multiple sexual partners or as a result of the loosening of surveillance of selective behaviors by the use of psychoactive substances (PAS)^(5-8)^. According to the Ministry of Health (MoH), in 2020, there was an increase in the AIDS detection rate among young people aged 15 to 24 years, with 33.2 cases/100,000 inhabitants, compared to 2010, which registered 27.2 cases/ 100,000 inhabitants. The highest incidence was observed in the 25-29 age group, with 43.2 cases/100,000 inhabitants^(4)^.

Thus, challenges and deficiencies persist in the formulation and implementation of public policies on STIs in Brazil that become effective and feasible in the different contexts of health care^(4)^ and that are able to reach different social groups and embrace their peculiarities, like young undergraduate students. This is because the behavioral and social changes, typical of youth, include the socially constituted context, which is limited to peculiarities related to entering the university^(5-6)^, which inserts them into a new membership group. In this new socio-relational reality, they begin to socially share content about daily life, such as STIs^(7-9)^.

Thus, it was decided to adopt the Social Representation Theory (SRT)^(9-11)^ framework. The field of study of SRT allows revealing the contents, structure and internal organization of social representations (SR). In this regard, SR are being conceived as a mechanism of “symbolization and meaning”, seen as a form of practical knowledge that links a subject to the social object, which has a diversity of natures (social, material or ideal)^(9)^. Such a perspective can be appropriated by nursing, due to the range of social phenomena that the theory reaches, such as the vulnerable sexual practices and behaviors of young undergraduate students to STIs.

It should be mentioned that the genesis of an SR occurs through two formative socio-cognitive processes capable of transforming what was considered unfamiliar into something familiar to social actors, namely: 1) anchorage: the social subject, faced with an unknown object, searches in his memory for related contents he knows, and transforms them, approaches the object as a “prototype”, comparing it so that the new is assimilated from something already existing; and 2) objectification: an abstract concept of reality, which was previously unknown, is reproduced, transforming it into a visible, concrete, palpable and tangible level^(9)^.

Faced with the problem presented, the need for this investigation is justified, since young people make up a population group that is more vulnerable to the occurrence of STIs^(2)^. Thus, identifying how young undergraduate students elaborate representational constructs regarding STIs can support new reflections on care for this group, through prevention and health promotion actions in the sexual and reproductive sphere.

## OBJECTIVE

To analyze the SR on IST prepared by young undergraduate students.

## METHODS

### Ethical aspects

All ethical and legal aspects of research involving human beings were met, with the matrix investigation entitled “*Saberes e práticas de prevenção de infecções sexualmente transmissíveis em uma perspectiva de gênero*”, submitted and approved to the Research Ethics Committee. Participant anonymity was ensured, using alphanumeric codes (MI1 and WI1). M refers to men, and W, to women, in addition to I, to interviewee, followed by the approach sequence number.

### Study design

This is a descriptive investigation, with a qualitative design, having as a theoretical-methodological framework the structural approach of SRT^(12)^, discussed in the light of (inter)national scientific evidence on the representational object. This text was prepared according to the Consolidated criteria for REporting Qualitative research (COREQ).

### Setting

The research setting was a public university located in the city of Rio de Janeiro (RJ), Brazil, whose choice made it possible to concentrate a number of undergraduate students from different areas of knowledge and with common characteristics, allowing to assume the existence of a social group.

### Data source

Sampling was non-probabilistic, for convenience, consisting of 160 undergraduate students of both sexes. Data saturation criteria were not adopted, since the recommendations of the SRT structural approach were met (n≥100)^(12)^. Undergraduate students regularly enrolled in the institution, aged between 18-29 years, sexually active and present in the academic units during the data collection period, were included. Undergraduate students who were absent due to medical leave or withdrawal from enrollment were excluded.

### Methodological procedures

The data collection instrument (DCI) was structured in: 1) sociodemographic characterization questionnaire, sexual practices and prevention with variables (sex, age, skin color, marital status, type of sexual partnership, condom use and negotiation of condom); 2) form for gathering free evocations of non-hierarchical words to the inducing term “STD”.

### Data collection and organization

The DCI was applied by a single researcher, trained in the collection of data from the structural approach of SRT, in the second semester of 2019, with an approximate duration of between 15 and 20 minutes. The free evocation technique is characterized by the production of words or expressions in response to inducing stimuli previously defined by the researcher, in order to identify representational contents and their structuring related to a social phenomenon^(10)^. It is noteworthy that it was not necessary to carry out any pilot test or familiarize participants with the data production technique, since the social group chosen was composed of young undergraduate students, a situation that provides them with experiences related to themes involving sexual practices and STIs.

Thus, undergraduate students were asked to mention the first five words that came to mind when they heard the inducing term “STD”. The adoption of the STD acronym is justified, as it is the expression most socialized and recognized by young people for the identification of STIs. Words and expressions were recorded on a form in the sequence in which they were mentioned. It should be noted that all undergraduate students were approached in common areas, invited to participate and clarified about the purpose and instruments of the research, having agreed to participate in the investigation.

### Data analysis

Sociodemographic characterization data and STI prevention practices were consolidated in the software Statistical Package for Social Sciences (SPSS), version 28, and treated using descriptive statistics. The evoked cognems were standardized, using semantic and lexicographical criteria that culminated in the *corpus*, which, in turn, underwent prototypical analysis. This type of analysis is based on the assumption that the representational contents that are important in their structure are more prototypical, i.e., they are more accessible to social actors’ awareness^(12)^.

The *corpus* was processed with the aid of software *Ensemble de Programmes Permettant l’analyse des Evocations* (EVOC) version 2005, which calculated the frequency of occurrences of each word, the weighted average of occurrence according to the order of evocation and the average of the weighted average orders of the set of words, providing, at the end, the Four-Quadrant Chart that configures the Four-Quadrant Chart technique, constituted according to Vergès^(9)^. In this framework, cognems were allocated into four quadrants, according to the frequency and order of evocation, in order to identify, in the upper left quadrant (ULQ), the central nucleus (CN); in the lower left (LLQ), the contrast elements; and in the upper right (URQ) and lower right (LRQ), the first and second peripheries, respectively.

In search of obtaining another indicative of the voked cognems’ centrality, a similarity analysis was carried out, which allowed to identify indices of co-occurrence of connection between the cognems, mentioned simultaneously by the same participant^(10-11)^. This type of analysis constitutes a procedure used in the structural perspective of the SR to investigate the number of connections that a word or expression has with other words in the SR^(12)^. Connections are established by calculating similarity indices between the most frequently evoked elements. The result is expressed in a maximum similarity tree that graphically synthesizes the set of existing connections between the group’s representational contents^(13)^.

For this purpose, the *corpus* was analyzed with the help of Microsoft^®^ Office Excel for Windows 2016, based on the evocations of participants by mentioning at least two cognems present in the Four-Quadrant Chart.

## RESULTS

Participants were undergraduate students, equally divided by sex, 80 women and 80 men. Among them, 122 (76.25%) were aged between 18 and 23 years and self-declared white skin color (90; 56.25%).

The characterization of sexual practices and STI prevention according to the frequency of answers was: they did not have a steady boyfriend or partner (76; 47.50%); reported using condoms in all sexual relations (85; 53.13%); had sex with a steady partner (118; 73.75%); used condoms with a fixed partnership (52; 32.50%); had sex with a casual partner (76; 47.50%); always used a condom with a casual partner (42; 26.25%); and did not negotiate condom use (74; 46.25%).

In the prototypical analysis, 748 evoked words-expressions were counted, 71 of which were different. Minimum frequency, 11, intermediate frequency, 24 and Rang 2.40 were adopted as parameters, which made up the Four-Quadrant Chart (Chart 1).

**Chart 1:** Four-Quadrant Chart for the inducing term “STD” among young undergraduate students at a university in Rio de Janeiro, 2023

AOE	< 2.4			≥ 2.4		
**Mean freq**.	**Evoked term**	**Freq.**	**AOE**	**Evoked term**	**Freq.**	**AOE**
≥ 24	AIDS	67	1,821	Syphilis	65	2.462
	Disease	48	2,292	Sex	52	2.481
	HIV	46	2,217	Condom	50	2.840
				Gonorrhea	46	3.283
				Prevention	36	3.194
< 24	Infection	11	2,000	Carelessness	22	2.955
				HPV	22	3.227
				Herpes	22	3.273
				Unawareness	17	3.353
				Treatment	15	3.533
				Fear	14	3.714
				Unprotected-sex	12	3.000
				Danger	11	2.727
				Contamination	11	3.182
				Candidiasis	11	4.091

*Note: n = 160; Min Fr = 11; Intermediate Fr = 24; Rang = 2.40. Content extracted from software EVOC 2005.*

The elements that constitute the likely CN, defined as the most important in terms of salience and order of evocation, located in the ULQ, were the terms AIDS, disease and HIV. In the contrast zone (LLQ), only cognem infection emerged. In the first periphery (URQ), cognems syphilis, sex, condoms, gonorrhea and prevention are found. Due to the high frequency, a term positioned in the first periphery may be a candidate for the CN potential^(10)^, and in this condition is cognem syphilis. Thus, hypothetically, this cognem shows signs of possible centrality as a representational construct. The second periphery (LRQ) presented cognems carelessness, HPV, herpes, unawareness, treatment, fear, unprotected-sex, danger, contamination and candidiasis.

**Figure 1:**
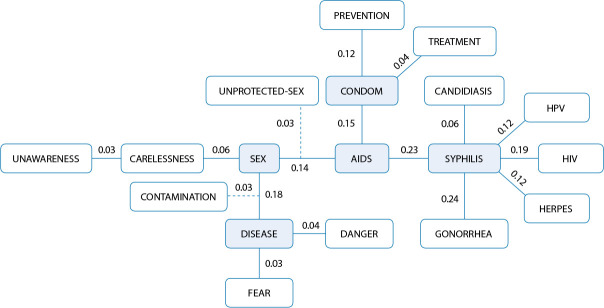
Maximum similarity tree referring to the evocations of the inducing term STD (N=153), Brazil, 2023

In Figure 1, the maximum similarity tree is presented, which allows the graphic demonstration of existing connections between the cognems present in Chart 1. The tree is constituted by five nuclei of meaning, organized by cognems syphilis, AIDS, condom, sex and disease, the first one, from right to left, shows that syphilis has six links, with gonorrhea (0.24), AIDS (0.23), HIV (0.19), herpes (0.12), HPV (0.12) and candidiasis (0.06), i.e., they are the most relevant connections in terms of quantity and binding strength. This result shows that the hypothesis of cognem syphilis may also have the possibility of being central, which was confirmed through the similarity tree. The second shows that AIDS establishes four links, with syphilis (0.23), condoms (0.15), sex (0.14) and unprotected-sex (0.03). Still in this chunk, condoms have three links, with AIDS (0.15), prevention (0.12) and treatment (0.04). The third shows that sex has three links, with disease (0.18), AIDS (014) and carelessness (0.06). The fourth shows that disease has four links, with sex (0.18), danger (0.04), fear (0.03) and contamination (0.03).

## DISCUSSION

The results of sociodemographic characterization and sexual practices presented were similar to those found in other investigations carried out with undergraduate students^(4-6,14)^.

The contents and the representational structure are compatible with the group’s level of academic training. It appears that, in SR, elements of reified universe are incorporated, thus building a concept about STIs. Information is also listed on the forms of transmission, preventive measures and especially the most common diseases in this scenario. Another aspect to be highlighted is the structure of the group’s thinking, evidenced in the similarity tree.

Starting from the nucleus of meaning formed by cognem AIDS, which is shown to be the maximum representative of this nosological picture in the group’s conception, there are three axes: one containing information about the forms of contamination, including those of a behavioral nature (unawareness, carelessness), highlighting the main forms of transmission of etiological agents among social actors (sex and unprotected-sex); a second axis, highlighting the most appropriate prevention method; and a third, in which they list the main diseases in the context of STIs. It is important to emphasize that this last axis reflects the knowledge they have about the vulnerability imposed by HIV and AIDS for the occurrence of other STIs.

Therefore, regarding the structure of representation, the central system points out that, for young undergraduate students, STIs comprise a set of diseases, with AIDS and HIV infection being more salient. Thus, AIDS is recognized as an STI as well as HIV, an element linked to it, translating the perception of the difference between being a carrier of the virus and the development of AIDS as a disease itself.

Therefore, it is considered that the group establishes a relationship between HIV and AIDS, i.e., when it reaches a more advanced stage of infection, it leads to acquired immunodeficiency syndrome (AIDS)^(10)^. Therefore, the group in question understands STIs as old and new diseases, denoting the conceptual dimension of SR^(9)^.

It is believed that the contrast zone presents complementary content from the periphery and the CN, based on cognem infection, as a specification of the type of disease or, yet, another way of conceptualizing STIs. Therefore, the cognitive process of construction, symbolization and meaning of the representational object by the group is highlighted considering the conceptual dimension, likewise observed in this quadrant.

It is verified that the first’s and second peripheries’ elements reveal knowledge (about transmission and types of diseases), behaviors (carelessness, unawareness) and practices that must be adopted for prevention (condoms and treatment). In other words, for the group, STIs are diseases acquired mainly through sexual intercourse, exemplified by syphilis and gonorrhea, which can be prevented with the use of condoms, recognized in the image of a condom. There are, therefore, two dimensions present in the first periphery: conceptual, since all elements translate into knowledge about STIs; and imagery, due to elements sex and condoms that translate, at the same time, a practice and an iconic expression of the studied object.

In the second periphery, it is observed that elements HPV, herpes, unawareness, treatment, contamination, candidiasis and unprotected-sex are inserted in a conceptual dimension of STIs; carelessness and unawareness, in the behavioral dimension; fear and danger, in the affective-attitudinal dimension, expressing associated judgments and affective expressions towards STIs. Thus, it is noticed that STIs arouse fear in young undergraduate students associated with unawareness, and reflect, at the same time, knowledge about prevention measures, treatment and forms of contamination.

Thus, it can be seen that sexual intercourse is recognized by the group as the main route of transmission of STIs detected by undergraduate students, emphasizing how necessary are the prevention measures and the efforts of early diagnosis of these infections, demanding educational practices as strategies of action^(14-17)^. In fact, through cognem unawareness, the group probably recognizes it as an element of vulnerability for the population, as it presents gaps in knowledge on the subject, which transits between knowing and not knowing what STIs are, how to prevent them, diagnose them, treat them and how to live with them, including the feelings that arouse throughout the process.

Despite this, some STIs (syphilis, gonorrhea, HPV, herpes, HIV, AIDS) were identified, demonstrating, at the same time, that the group is aware of which are prevalent, mainly due to access to information widely available in schools, in the media, in widely disseminated publications, in everyday conversations and also in protection and prevention campaigns published in the media, organized and available on the Ministry of Health website, such as the 2020 STI Prevention Campaign and many others^(18)^.

Furthermore, despite mentioning some STIs in relation to prevention, condoms and unprotected-sex are mentioned, pointing to the recognition of the need to use condoms and their absence associated with the occurrence of STIs present on the Four-Quadrant Chart’s periphery. This representational composition of STIs requires questioning the reasons that lead or not to the adoption of safer sexual practices as well as the situations of vulnerability that are associated with the abandonment of condom use. These expressions of vulnerability are expressed in the guarantee of sexual pleasure, in the stability of relationships, in female submission behaviors, in trust with partners and in the understanding of condoms as a contraceptive barrier, which can be replaced by other methods without compromising sexual pleasure^(18)^.

Thus, although one cannot conceive of a unicausal relationship between STIs and condom use, it is important to highlight that some common-sense conceptions can be interpreted as protective, but, in fact, function as vulnerability factors. Moreover, as much as young undergraduate students know and mention some IST, this knowledge is not enough to prevent exposure to these harms, as evidenced in the social characterization of the group in which a portion does not systematically use condoms. On the other hand, it can be assumed that it may also be related to the affective-attitudinal representational dimensions that STIs awaken, such as fear and danger.

It is noteworthy that the fear of contamination immobilizes and prevents the adoption of preventive strategies in everyday sexual practice. Likewise, such behavior may occur in the search for guarantees and pleasure without using condoms as well as the apparent solidity of affective relationships, in which condoms are judged as expendable.

In this sense, the results of some current studies are similar and illustrate the plot presented in the representational structure, emphasizing the hegemonic masculinity and the conceptions regarding unprotected sex. Also, issues related to gender and the search for pleasure or romantic love are highlighted, which lead to the abandonment of condoms, without paying attention to the risk of contracting diseases and infections, leaving young people exposed to the experience of sexuality, vulnerable to STIs and unprotected sex, reinforced by the social imaginary of a romantic love that would work as protection^(9-10,15,19-20)^.

It is clear, then, that STI prevention resides in the challenge of promoting the representational change of STIs, of other objects that are involved in this plot, i.e., in the SR of sex, condoms, among others. This assertion is based on the fact that only in the peripheral system is prevention mentioned, highlighting as central to the structure of this representation only the recognition of STIs as a disease and their association with HIV and AIDS. Promoting a change in this SR network could allow modifications in the elements used for prevention, changing its status to CN. This could also be reflected in changes in practices^(21)^, insofar as the change in a social practice is conditioned, in certain situations, by the change in the structuring SR, i.e., the one that supports the subgroups’ representational constructs.

The maximum similarity tree allows confirming the possibility of centrality of some constituent elements of SR. It is observed that some cognems have reaffirmed the possibility of indicating centrality, since they are in the CN in the prototypical analysis and have established a greater number of connections with other elements, in addition to presenting high similarity rates, as is the case of AIDS and disease, already present in the ULQ. But it also reinforces the hypothesis of centrality in relation to cognem syphilis, with high frequency in the Four-Quadrant Chart’s first periphery. Terms condom, sex and syphilis, present in the first periphery, also present these characteristics, but are not central in the prototypical analysis, and can be considered overactivated peripheral elements.

It is worth reflecting that even the data related to sexual practices, when analyzed in a triangulated way to the results of SR, justify the presence of cognem condom as an overactivated peripheral element, given its centrality behavior by the quantity and strength of connections it establishes with other elements. It is possible to infer that, due to the fact that participants were undergraduate students, the group’s social thinking about STIs is strongly anchored in the knowledge they have about condoms, referring to their practical utility in terms of prevention (use) and treatment of diseases (use/disuse), although it does not imply prevention practice on the part of some participants, as observed in the group profile.

Thus, it appears that the first chunk presents syphilis as the element with the most connections and with the greatest connection strengths with gonorrhea, AIDS and HIV, which reinforces the recognition of the current overview of public health in the country, with HIV/syphilis co-infection a considerable frequency, evidencing the vulnerability imposed by HIV infection, as previously mentioned. Moreover, this result is also related to the fact that syphilis is an old STI, prior to the AIDS pandemic^(21)^, which explains why it was mentioned more often and established many connections in the maximum similarity tree, including with other STIs.

In this regard, current statistics warn of a syphilis epidemic in Brazil, which draws attention to the possibility of co-infections with other STIs and to the vulnerability of people living with HIV, as the prevalence of syphilis is disseminated among the different population segments. Furthermore, co-infection with other STIs can lead to more aggressive cases and complications, especially for those who experience AIDS, such as early meningovascular syphilis and herpes, with more lasting, painful and atypical lesions. In addition to this, candidiasis, although not considered an STI, is an endogenous infection that can be confused with an STI, and is among the predisposing factors for HIV infection^(2-4)^.

The second chunk of similarity analysis deepens the context related to sexual practices, where the group demonstrates knowledge about the importance of using condoms as a method of preventing STIs, with emphasis on AIDS and syphilis, possibly because they are the most widespread infections in the media^(15-16)^.

The link between condoms and treatment deserves to be highlighted, as it points to the group’s perception of sexual care. On the one hand, this connection points to the possibility that the group does not consider any treatment necessary if condoms are used, but, on the other hand, it may recognize the option of adhering to treatments, in case of contamination, instead of using condoms. This can be observed in some studies, especially after the advent of antiretroviral therapy, which brought greater quality and life expectancy for people living with HIV and ruled out the possibility of death, as it was observed in the 80s and 90s. On the other hand, it converges to a greater vulnerability of young people who stop using condoms, based on the possibility of treatment^(9-10)^.

The third chunk focuses on the main concepts that participants associate with STIs, reinforcing the group’s recognition of the fact that STIs are diseases acquired mainly through sexual practice, either because of carelessness in adopting safe sexual practices, or because of possible unawareness about prevention measures.

This perspective is discussed in view of naturalization of young people in relation to sexual health, when observing lack of attention in relation to carrying out periodic preventive examinations for screening and early diagnosis. Moreover, it is opportune to consider the possibility of preventing STIs in line with safer sexual practices and efforts aimed at controlling vulnerabilities, through their understanding. In this context, it is added that many young people have insufficient knowledge about STIs, being closer to the most publicized ones, such as HIV, and are vulnerable to other^(6,15)^.

The tree’s fourth chunk allows understanding the emotional impact that STIs can cause and the connections that communicate important information to affective elements associated with these diseases. Participants demonstrate fear of acquiring an STI, because they know what it means, even if superficially.

Fear, in fact, can originate from several aspects involving the context of STIs. One of them may be related to the form of contamination, such as sexual violence and rape, which lead people to develop a fear of reporting for reasons related to humiliation, lack of legal knowledge about the act and the feeling of guilt. Another aspect may be related to the fear of rejection by sexual partners and the ability to bear children^(16-17)^.

Given the above, there is an important information gap to be worked on by health professionals, whose greatest impact is based on the feelings and affective dimension that STIs awaken in young undergraduate students and that have the potential to reinforce and/or immobilize preventive actions. Additionally, the process of naturalization of sexual practices conditions their non-association with diseases that can be acquired by these practices.

The investigated young people demonstrate a certain reified knowledge about STIs, which does not translate into the direct adoption of safe sexual practices, requiring the work of health educators in Higher Education Institutions (HEIs) such as universities. These have the ability to provide undergraduate students with up-to-date, sufficient information corresponding to their social realities, leading them to the process of reflection about sexual behavior, minimizing doubts, preventing injuries related to sexual health, STIs and giving the university the status of a health promoting institution^(15,22-23)^. Besides that, health professionals’ performance, such as nurses, is perceived, mainly in Primary Care, with educational actions aimed at STI/AIDS prevention, when sharing professional practices of an informative, educational and communicational nature that can be directed to this scenario^(9)^.

SR on STIs show that young undergraduate students built a network of meanings that approximates the reified knowledge on the subject as well as their alternatives for meaning safer sexual behaviors, with a view to preventing STIs^(24-25)^. However, the naturalization of sexual practice and the affective-attitudinal components of SR of STIs seem to act to prevent the adoption of safer sexual behaviors^(11-13)^. Educational work requires, therefore, the recognition of this network of determinations and an action that seeks to make a greater association between SR and practices.

### Study limitations

A limitation of this study is that it was carried out in a single HEI. However, the results are related to the adopted methodological outline, and point out the peculiarities of a socially constituted group. Other studies using the SR of young undergraduate students about STIs and prevention practices, which could add other approaches, would be opportune for a greater deepening of this research theme.

### Contributions to nursing, health, or public policies

The need to promote actions aimed at sexual health is evident, with emphasis on carrying out educational actions in health that are inclusive of the young triad, sexual partners and social group, problematizing and contextualized to the reality experienced by these social actors. Thus, the importance of their knowledge regarding AIDS and syphilis as the main STIs represented by the group is highlighted as well as their awareness of the occurrence of other associated or isolated STIs, which require safe prevention strategies, such as consistent use of barrier methods and safer sexual behaviors.

## FINAL CONSIDERATIONS

SR of STIs for young undergraduate students demonstrates social thinking based on specific knowledge, in order to recognize STIs as diseases that require barrier prevention measures aimed at condoms. Thus, they associate the danger of adopting unsafe sexual behaviors and practices (unprotected sex) with the possibility of exposure to STIs. Still, they associate images that illustrate this universe (sex and condoms) and affective elements (fear), which can immobilize and impede prevention actions.

Moreover, AIDS and syphilis stand out in the recognition and representational construct of STIs, possibly because they are widely disseminated in the media and academia, through protection and prevention campaigns against STIs and by focusing on old and new infectious diseases that result from the adoption of vulnerable sexual practices as the main means of contamination, even though they are avoidable.
